# Substance P and Neurokinin-1 Receptor System in Thyroid Cancer: Potential Targets for New Molecular Therapies

**DOI:** 10.3390/jcm12196409

**Published:** 2023-10-09

**Authors:** Inmaculada Isorna, Miguel Ángel González-Moles, Miguel Muñoz, Francisco Esteban

**Affiliations:** 1 Department of Otorhinolaryngology, Hospital Universitario Virgen del Rocio, 41013 Seville, Spain; inmaisorna@gmail.com (I.I.); festebano@gmail.com (F.E.); 2 School of Dentistry, University of Granada, 18010 Granada, Spain; magonzal@ugr.es; 3 Research Laboratory on Neuropeptides, Institute of Biomedicine of Seville (IBiS), 41013 Seville, Spain; 4 School of Medicine, University of Seville, 41009 Seville, Spain

**Keywords:** neurokin-1 receptor, substance P, tachykinin, thyroid gland, thyroid cancer

## Abstract

In recent years, numerous approaches have been developed to comprehend the molecular alterations underlying thyroid cancer (TC) oncogenesis and explore novel therapeutic strategies for TC. It is now well established that the neurokinin-1 receptor (NK-1R) is overexpressed in cancer cells and that NK-1R is essential for the viability of cancer cells. The binding of substance P (SP) to NK-1R in neoplastic cells plays a pivotal role in cancer progression by promoting neoplastic cell growth, protecting tumor cells from apoptosis, triggering invasion and metastasis through the enhanced migration of cancer cells, and stimulating endothelial cell proliferation for tumor angiogenesis. Remarkably, all types of human TC (papillary, follicular, medullary, anaplastic), as well as metastatic lesions, exhibit the overexpression of SP and NK-1R compared to the normal thyroid gland. TC cells synthesize and release SP, which exerts its multiple functions through autocrine, paracrine, intracrine, and neuroendocrine processes, including the regulation of tumor burden. Consequently, the secretion of SP from TC results in increased SP levels in plasma, which are significantly higher in TC patients compared to controls. Additionally, NK-1R antagonists have demonstrated a dose-dependent antitumor action. They impair cancer cell proliferation on one side and induce apoptosis of tumor cells on the other side. Furthermore, it has been demonstrated that NK-1R antagonists inhibit neoplastic cell migration, thereby impairing both invasiveness and metastatic abilities, as well as angiogenesis. Given the consistent overexpression of NK-1R in all types of TC, targeting this receptor represents a promising therapeutic approach for TC. Therefore, NK-1R antagonists, such as the drug aprepitant, may represent novel drugs for TC treatment.

## 1. Introduction

Thyroid cancer (TC) is the most common endocrine malignant neoplasm [1]. Papillary and follicular morphologic types, accounting for over 90% of all TC cases, range from indolent localized papillary carcinomas to aggressive anaplastic neoplasms [2]. Thyroid cancers of follicular cell origin from epithelial cells comprise various subtypes, including papillary (PTC, 80%), follicular (FTC, 11%), and rarer variants such as poorly differentiated TC (PDTC), Hürthle cell TC, insular carcinoma, follicular variant of PTC, and tall cell carcinoma [3]. Medullary TC (MTC), derived from calcitonin-producing parafollicular cells (C cells), represents 5–10% of all TCs [4], while anaplastic TC (ATC) is an extremely aggressive neoplasm, observed in only 2% of patients, with a survival rate of less than six months. Lymphomas and sarcomas are rare subtypes reported in the thyroid gland [5]. In recent years, TC incidence has been rapidly increasing worldwide, predominantly among women, and PTC is projected to become the third-most common cancer in women [6].

Considerable efforts have been devoted to investigating the molecular mechanisms underlying TC oncogenesis in the past decade [7]. Current management options center around thyroidectomy and radioactive iodine (RAI) treatments, the specifics of which are determined by individual risk stratification. Recurrences are managed surgically, along with radioactive iodine and radiotherapy [8]. There is a need to investigate other potential therapeutic molecular interventions, particularly in recurrent and metastatic TC. Neurokinin-1 receptor (NK-1R) antagonists have shown potential in TC treatment, as the substance P (SP)/NK-1R system is expressed in human TC.

The TAC1 gene encodes the SP peptide, which belongs to the tachykinin family, including hemokinin-1 (HK-1), neurokinin A (NKA), and NKB. Tachykinins exert various biological functions upon binding to NK-1R, NK-2R, and/or NK-3R. NK-1R, encoded by the TACR1 gene, exhibits preferential affinity for SP/HK-1, while NKA and NKB preferentially bind to NK-2R and NK-3R, respectively [9,10]. NK-1R is a member of the G protein-coupled receptor family and consists of three intracellular and three extracellular loops, with a possible additional fourth loop [9]. Two isoforms of NK-1R exist: full-length and truncated. The truncated isoform, lacking the last 96 amino acids of the C-terminus, is overexpressed in neoplastic cells and remains continuously present in the cell membrane without internalization [11,12,13]. SP is widely distributed throughout the body and regulates numerous biological functions, including pain, neurogenic inflammation, and mitogenesis.

NK-1R is overexpressed in neoplastic cells, with truncated NK-1R being more prevalent than the full-length molecule [12,13]. NK-1R has been reported to be essential for cancer cells [14], as SP binding to NK-1R promotes cancer cell proliferation, suppresses apoptosis, activates angiogenesis, and enhances the migratory capacity of neoplastic cells, thereby increasing invasiveness and metastatic potential [15]. Several studies have demonstrated that NK-1R antagonists counteract these SP-mediated effects, inhibiting cancer cell proliferation and promoting apoptosis in human tumor cells [12,15,16,17,18,19]. Considering the significance of the SP/NK-1R system in cancer progression, NK-1R represents a potential target for cancer treatment, with NK-1R antagonists being considered as broad-spectrum antineoplastic agents [19]. In light of the overexpression of the SP/NK-1R system in TC, we propose the use of NK-1R antagonists in TC patients. Therefore, the primary objective of this paper is to review the existing data on SP and NK-1R in TC and provide a basis for future studies that could justify the utilization of NK-1R antagonists in TC patients [16,20].

## 2. Substance P and Neurokinin-1 Receptor in Thyroid Cancer

**Figure 1:**
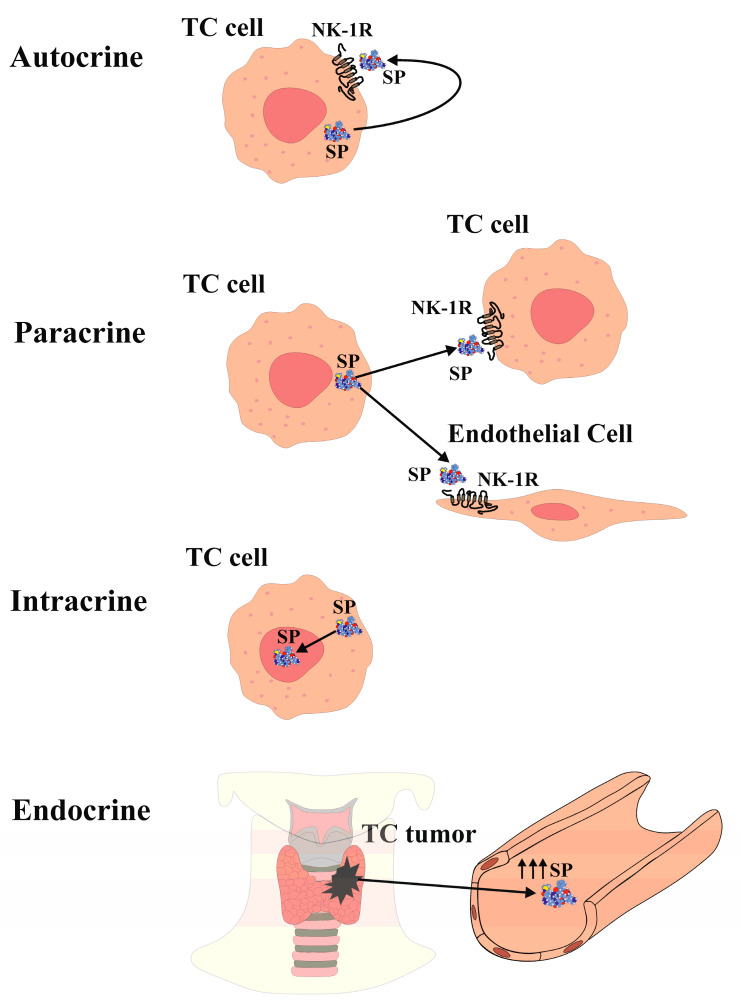
SP functions in TC.

The expression of SP and NK-1R in the thyroid gland and thyroid cancer (TC) has been investigated for over 40 years. Previous studies reported elevated blood levels of SP in a patient with medullary TC [21]. Immunohistochemistry analysis demonstrated SP expression in only one out of twenty-seven medullary TC cases in one study [22], while another study detected NK-1R expression in ten out of twelve medullary TC cases [23]. However, recent research has demonstrated the presence of both SP and NK-1R in normal thyroid tissue as well as in samples representing the four main types of TC (anaplastic, follicular, medullary, and papillary) [20]. Both SP and NK-1R were found in all TC and normal thyroid samples examined, and their expression was higher in TC tissue compared to healthy thyroid tissue. Notably, both SP and NK-1R were expressed in all normal and medullary TC samples [20]. Furthermore, a case-control study involving 31 healthy volunteers and 31 TC patients reported higher plasma levels of SP in the tumor group compared to the control group, along with a stronger expression of NK-1R in TC specimens compared to the surrounding normal tissue [24]. The discrepancy in the results when comparing previous studies with the most recent ones, where the expression of SP and NK-1R in the TC samples was so low in previous papers whereas the recent studies reported an overexpression in 100% of all the samples studied, could probably be explained by the use of more specific antibodies and a much more refined technique. These refined immunodetection techniques have provided substantial evidence for the widespread presence of both NK-1R and SP, with elevated SP serum levels observed in TC patients compared to controls. Moreover, SP and NK-1R are expressed in the thyroid gland and are overexpressed in all types of TC [20,24]. Additionally, the presence of SP/NK-1R in the nucleus and cytoplasm of TC follicular cells aligns with findings in various human malignant neoplasms, such as keratocystic odontogenic tumors, gastric carcinoma, laryngeal squamous cell carcinoma, oral squamous cell carcinoma, small and nonsmall lung cancer, melanoma, and breast cancer [25,26,27,28,29,30,31,32,33]. SP has been detected within the nucleus of endothelial cells in FTC samples, as well as in the nucleus of endothelial cells and myocytes of fetal blood vessels, decidua, and trophoblast [34]. Furthermore, SP and NK-1R have been identified in the nucleus of stem cells [35]. These findings collectively demonstrate the presence of the SP/NK-1R system within the nucleus of TC cells, suggesting a role in regulating nuclear activity in an epigenetic manner, thereby influencing cell behavior [20,35]. The SP/NK-1R system in the limbic system of the central nervous system has also been implicated in regulating emotional behavior [36]. Furthermore, SP can be considered an epigenetic factor that modulates gene expression in TC cells, affecting processes such as cellular differentiation, cell cycle progression, angiogenesis, inflammation, and apoptosis, through interactions with various transcription factors and proto-oncogenes [37,38,39]. SP is observed in the cytoplasm of both normal and neoplastic TC follicular cells, indicating potential autocrine, paracrine, intracrine, and/or endocrine actions (Figure 1). Notably, NK-1R is present in the colloid of TC samples but not in normal thyroid tissue, possibly related to the higher turnover of neoplastic cells in TC compared to normal thyroid tissue [20].

At nanomolar concentrations, SP induces mitogenesis in many human neoplastic cells, which may explain the overexpression of its receptor [40,41,42]. Through NK-1R, SP activates various molecules in the mitogen-activated protein kinase (MAPK) cascade, leading to the translocation of extracellular signal-regulated kinases 1 and 2 (ERK1/2) into the nucleus, thereby promoting cell proliferation. The activation of the MAPK cascade requires the presence of a functional epidermal growth factor receptor (EGFR) kinase domain [43]. Another potential pathophysiological effect of SP on tumor cells is its antiapoptotic effect, preventing tumor cell death. SP binding to NK-1R enhances the phosphorylation and activity of protein kinase B (Akt), exerting an antiapoptotic effect [44].

The release of SP from TC cells suggests both an autocrine action (Figure 1) of SP binding to NK-1R that is overexpressed in TC cells and induces TC cell proliferation, and a paracrine action (Figure 1) of the peptide on NK-1R-expressing endothelial cells, potentially inducing proliferation and promoting neovascularization within and around the tumor, thereby enhancing TC growth [15,45]. Furthermore, NK-1R is found in blood vessels, and its expression increases during neoangiogenesis [23].

The term “intracrine” refers to molecules that act within a cell to regulate intracellular events [46]. Peptide/protein hormones that exhibit intracellular function are also known as “intracrine”. Given the localization of SP in the cytoplasm and in the nucleus of TC cells [20], SP is believed to exhibit an intracrine (nucleocrine) function in TC cells (Figure 1). While peptides typically act as endocrine, autocrine, or paracrine by binding to receptors on the cell surface, SP in TC cells also acts through intracrine mechanisms. Notably, SP is expressed within the nucleus of TC cells, whereas NK-1R expression in the nucleus is exceptional. This raises the question of whether SP directly binds to the DNA in TC cells. It is known that TATA-box binding protein (TBP) binds to the minor groove of DNA at the TATA box sequence, producing a large-scale deformation in DNA and initiating transcription. Moreover, the binding of small molecules or proteins to DNA often leads to DNA deformation, and that DNA folding is facilitated by the projection of two phenylalanine residues from the TPB into the minor groove [47]. Interestingly, SP contains two phenylalanine residues at its C-terminus [48].

Furthermore, SP can be released into the bloodstream from the TC mass, suggesting an endocrine action (Figure 1), leading to elevated SP plasma levels. This is supported by the observation of high SP levels in the plasma of a patient with medullary TC [21]. Additionally, SP serum levels were higher in TC patients compared to healthy volunteers [24]. The elevated blood levels of SP may contribute to the development of paraneoplastic syndromes, such as thrombosis, pruritus, emotional stress, and malnutrition. Platelets express NK-1R, and SP can induce thrombosis, whereas NK-1R antagonists have been shown to reduce thrombus formation [49]. Anxiety and depression have also been associated with high plasma SP levels, suggesting that SP release from TC masses may contribute to depression by establishing a cross-talk between the limbic system (emotional stress) and TC, creating a reciprocal relationship [36]. Furthermore, elevated SP levels may be implicated in pruritus, as SP itself can induce pruritus, and NK-1R antagonists have shown efficacy in alleviating this symptom [50]. Experimental data suggest that SP and NK-1R are involved via different mechanisms in TC oncogenesis (Figure 1). The release of SP may induce mitogenesis in TC cells, enhance an antiapoptotic effect, promote TC angiogenesis, and stimulate TC cell migration, thereby facilitating invasion and metastasis. Moreover, SP levels are elevated in TC patients, NK-1R is overexpressed in TC cells, and NK-1R is essential for the viability of TC cells.

## 3. NK-1R Antagonists as Potential New Therapeutic Agents for Molecular Therapy

NK-1R antagonists are a group of specific nonpeptide molecules that share similar stereochemical features and are resistant to degradation by peptidases, making them suitable candidates for thyroid cancer (TC) therapy. NK-1R antagonist agents and drugs are useful in tumors that overexpress NK-1R. In a concentration-dependent manner and after specific binding to NK-1R, NK-1R antagonists present numerous antitumor effects such as antiproliferative and proapoptotic, antiangiogenic, counteracting the Warburg effect, and anti-invasion and antimetastatic effects. Since it has been shown that all types of TC overexpress NK-1R, treatment with specific NK-1R antagonists would present the same therapeutic effects that have been observed in other tumor cell types.

### 3.1. Therapeutic Effect of NK-1R Antagonists as Antiproliferative and Proapoptotic Agents and Drugs in Cancer That Overexpress NK-1R

Currently, there are four NK-1R antagonists, namely L-733,060, L-732,138, CP-96,345, and the aprepitant drug (approved for chemotherapy-induced nausea/vomiting (CINV) in humans), which have demonstrated activity against cancer cells both in vitro and in vivo [16]. These NK-1R antagonists inhibit neoplastic cell proliferation [12,17,18,20] and induce apoptosis in several and different tumor cell lines in a concentration-dependent manner in NK-1R-overexpressing tumor cells [12,15,16]. Studies have shown that L-733,060 and the aprepitant drug increase the occurrence of apoptotic DNA features, leading to the cleavage of caspase-3 and poly (ADP-ribose) polymerase in hepatoblastoma and glioma cell lines [12,44]. L-733,060 also enhances apoptosis, inhibits the basal kinase activity of Akt, and induces similar effects on caspase-3 and poly (ADP-ribose) polymerase in glioma cells [44].

### 3.2. Therapeutic Effect of NK-1R Antagonists as Antiangiogenic Agents and Drugs in Cancer That Overexpress NK-1R

Angiogenesis plays a crucial role in cancer progression, as solid tumors require an efficient blood supply. NK-1R overexpression has been associated with angiogenesis, and NK-1R has been detected in intra- and perineoplastic blood vessels in various tumors [25]. SP directly stimulates angiogenesis by inducing endothelial cell proliferation. However, specific NK-1R antagonists can block SP-induced angiogenesis [51]. In a xenograft mouse model of hepatoblastoma, aprepitant administration at a dose of 80 mg/kg/day for 24 days resulted in a significant reduction in tumor growth and vascularized area within the tumor [12]. Furthermore, aprepitant has been reported to decrease vascular endothelial growth factor (VEGF) expression, a well-known marker of angiogenesis, in osteosarcoma cells [52]. These findings strongly support the potential use of aprepitant in TC to reduce or inhibit tumor angiogenesis.

### 3.3. Therapeutic Effect of NK-1R Antagonists Agents and Drugs in Counteracting the Warburg Effect in Cancer That Overexpress NK-1R

Neoplastic cells primarily rely on a high rate of glycolysis followed by lactic acid fermentation, a phenomenon known as the Warburg effect [53]. Studies have shown that SP promotes glycogen breakdown and increases the intracellular calcium concentration in glioblastoma cells overexpressing NK-1R. However, the NK-1R antagonist CP-96,345 has been found to counteract these effects [54]. It has been suggested that the glycolytic function is directly related to the number of NK-1R receptors expressed in each cell, with tumor cells exhibiting higher expression levels and increased glycolytic rates [15]. This therapeutic effect of NK-1R antagonists is particularly important, as there are currently no available drugs that specifically counteract the Warburg effect.

### 3.4. Therapeutic Effect of NK-1R Antagonists as Anti-invasion and Antimetastatic Agents and Drugs in Cancer That Overexpress NK-1R

Despite favorable survival rates in most TC patients, those with local invasion and metastasis often do not respond well to standard treatments and have a poor prognosis [55]. Tumor cell migration is critical for invasiveness and metastatic potential, with metastasis being the leading cause of cancer-related deaths [56]. SP, upon binding to NK-1R, induces changes in the cancer cell shape, including blebbing, which is essential for cancer cell spreading and infiltration [57]. Interestingly, U373MG astrocytoma tumor cells display significantly more rapid and transient membrane blebbing compared to nontumor cells [57]. Moreover, SP has been shown to increase the expression of matrix metalloproteinase (MMP-9) and vascular endothelial growth factor-C (VEGF-C), both of which are associated with vascular invasion and lymphatic metastasis. However, NK-1R antagonists L-733,060 and L-732,138 inhibit the expression of both VEGF-C and MMP-9 in endometrial adenocarcinoma cells, suggesting the potential reduction in invasiveness and metastatic capabilities [58]. Furthermore, SP promotes migration and induces the expression of MMP-2, MMP-9, VEGF-A, and VEGF receptor 1 (VEGFR1) in esophageal cancer cells, whereas aprepitant counteracts these effects [59]. The antimetastatic effect of aprepitant in TC could be attributed to its modulation of NF-κB and subsequent downregulation of its target genes, including VEGF-A, VEGF-C, VEGFR1, MMP-2, and MMP-9 [52,58,59].

## 4. Conclusions

An overexpression of SP and NK-1R and consequent autocrine, paracrine, and intracrine actions may have a role in TC oncogenesis. Furthermore, promoting TC cell proliferation, activating the Warburg effect, inhibiting apoptosis, stimulating angiogenesis, and facilitating cell migration may also contribute to an invasive and metastatic phenotype. The experimental data are compelling. A translation of these data to the design and implementation of clinical studies investigating SP and NK-1R as potential avenues for new molecular therapies is necessary for new drug development.

## Data Availability

No new data were created.
